# The complete mitochondrial genome of two-belt cardinal and striped cardinalfish *Apogonichthyoides taeniatus* (Cuvier, 1828) 

**DOI:** 10.1080/23802359.2021.1993102

**Published:** 2021-10-23

**Authors:** Min Yang, Jianling Huang, Xinshuai Li, Jinpeng Chen, Zewen Li, Shikai Chen, Xuezhu Li

**Affiliations:** a Joint Laboratory of Guangdong Province and Hong Kong Region on Marine Bioresource Conservation and Exploitation, College of Marine Sciences, South China Agricultural University, Guangzhou, China; b Guangdong Laboratory for Lingnan Modern Agriculture, Guangzhou, China

**Keywords:** *Apogonichthyoides taeniatus*, mitochondrial, genome

## Abstract

The complete mitochondrial DNA sequence of *Apogonichthyoides taeniatus* (Cuvier, 1828) is determined. The mitochondrial genome is 17,050 in length and has the same composition and gene order like most other vertebrates. The phylogenetic analysis based on 13 concatenated PCGs nucleotide sequences among 20 species showed that this species has high support with the sister branch *Jaydia lineata*. Our findings provide useful information for phylogenetic and evolutionary research of Kurtiformes species.

The two-belt cardinal and striped cardinalfish *Apogonichthyoides taeniatus* (Cuvier, 1828), belong to Apogoninae, Kurtiformes. It distributes generally in the Indian Ocean-western and South China Sea (Masuda et al. 1984). *A. taeniatus* generally inhabit coastal coral reefs and mangrove waters. The biology of *A. taeniatus* is poorly studied, with only morphological and behavioral studies reported (Thresher et al. 1984; Tortonese 1986). In the present study, we characterize the complete mitochondrial genome DNA (mtDNA) sequence of *A. taeniatus*.

*A. taeniatus* were collected from Hailing Island, Yangjiang city, Guangdong province, China (111°52′00″E, 21°40′00″N). The genomic DNA extracts from the standard phenol/chloroform method (Sambrook and Russel 2002). The method for preparing and sequencing the DNA library is referred to Zhu et al. (2017a, 2017b). Twenty-five primer pairs designed from the mitogenome of related species were used to amplify the *A. taeniatus* mitogenome (Supplementary Table 1). The obtained partial sequences of *A. taeniatus* were assembled using Geneious R11.1.5 (Kearse et al. 2012), and the final complete mitogenome was annotated based on the *Apogon semilineatus* mitogenome (GenBank: AP005996). The specimens and DNA were deposited at the College of Marine Sciences, South China Agricultural University (https://www.scau.edu.cn, Min Yang, yangmin2016@scau.edu.cn) under voucher number SCAU-AT-0404.

The complete mtDNA is 17, 050 bp in length (Genbank Accession number: MN699562), with a high A + T content (51.80%). A (27.85%), T (30.97%), C (19.18%), and G (21.99%) are in D-loop region. There are eight overlapping regions, ranging from 1 to 10 bp. And 13 intergenic regions exist in this genome, ranging from 1 to 35 bp. The gene content is equal to many other teleost mtDNA (Yagishita et al. 2009; Zhang et al. 2016; Zhu et al. 2017a), comprising 13 protein-coding genes (PCGs), 22 tRNAs, 2 rRNAs, and a control region.

Only one PCGs (*ND6*), and eight tRNA genes (Gln, Ala, Asn, Cys, Tyr, Ser, Glu, and Pro) are encoded by L strand, while two rRNA genes (12S rRNA and 16S rRNA), twelve PCGs (*ND1*, *ND2*, *COX1*, *COXII*, *ATP8*, *ATP6*, *COXIII*, *ND3*, *ND4L*, *ND5*, and *Cytb*), fourteen tRNA genes (Phe, Val, Leu, Ile, Met, Trp, Asp, Lys, Gly, Arg, His, Ser, Leu, and Thr), and a non-coding region (D-loop region) are encoded by H strand. Twelve of thirteen PCGs start with the representative initiation codon ATG, only *COX1* starts with GTG. Nine PCGs initial TAA and *ND2* use AGA as a stop codon, *ND6* use AGG as a stop codon, whereas *COXII*, *ND4*, and *Cytb* use incomplete stop codon, T-. It is analogously with that in *Toxotes chatareus*, *Elagatis bipinnulata*, and *Lutjanus carponotatus* (Yagishita et al. 2009; Ma et al. 2017; Kim et al. 2019).

There are 22 tRNAs genes, ranging from 66 bp (tRNA^Cys^) to 75 bp (tRNA^Leu^). The non-coding region is A + T enrichment region, which is related to transcription and replication (Clayton 1992). The non-coding region is located between tRNA^Pro^ and tRNA^Phe^ genes, is 1314 bp length with high A + T content (58.82%). All the tRNAs can be folded into the representative cloverleaf secondary structures.

To investigate phylogenetic relationships between Centrarchiformes, Lutjaniformes, Pempheriformes, and Kurtiformes, a phylogenetic tree is constructed by MEGA 7.0 based on 13 tandem PCGs nucleotide sequences with Maximum likelihood (ML) method and MtMam + I + G + F model (Kumar et al. 2016). Because both two species belong to Kurtiformes, *A. taeniatus* is grouped with *J. lineata* as the sister species (Figure 1). The genetic distance among Kurtiformes/Apogonidae species was also calculated basing on the complete mtDNA sequence by MEGA 7.0, and the results showed that the species in descending order of genetic distance from *A. taeniatus* were *P. trimaculatus*, *J. lineata*, *C. quinquelineatus*, *A. semilineatus*, *S. orbicularis*, *P. kaudemi* (Supplementary Table 2). The *A. taeniatus* mtDNA will provide additional genetic information for genetic analyses in future study.

**Figure 1.**
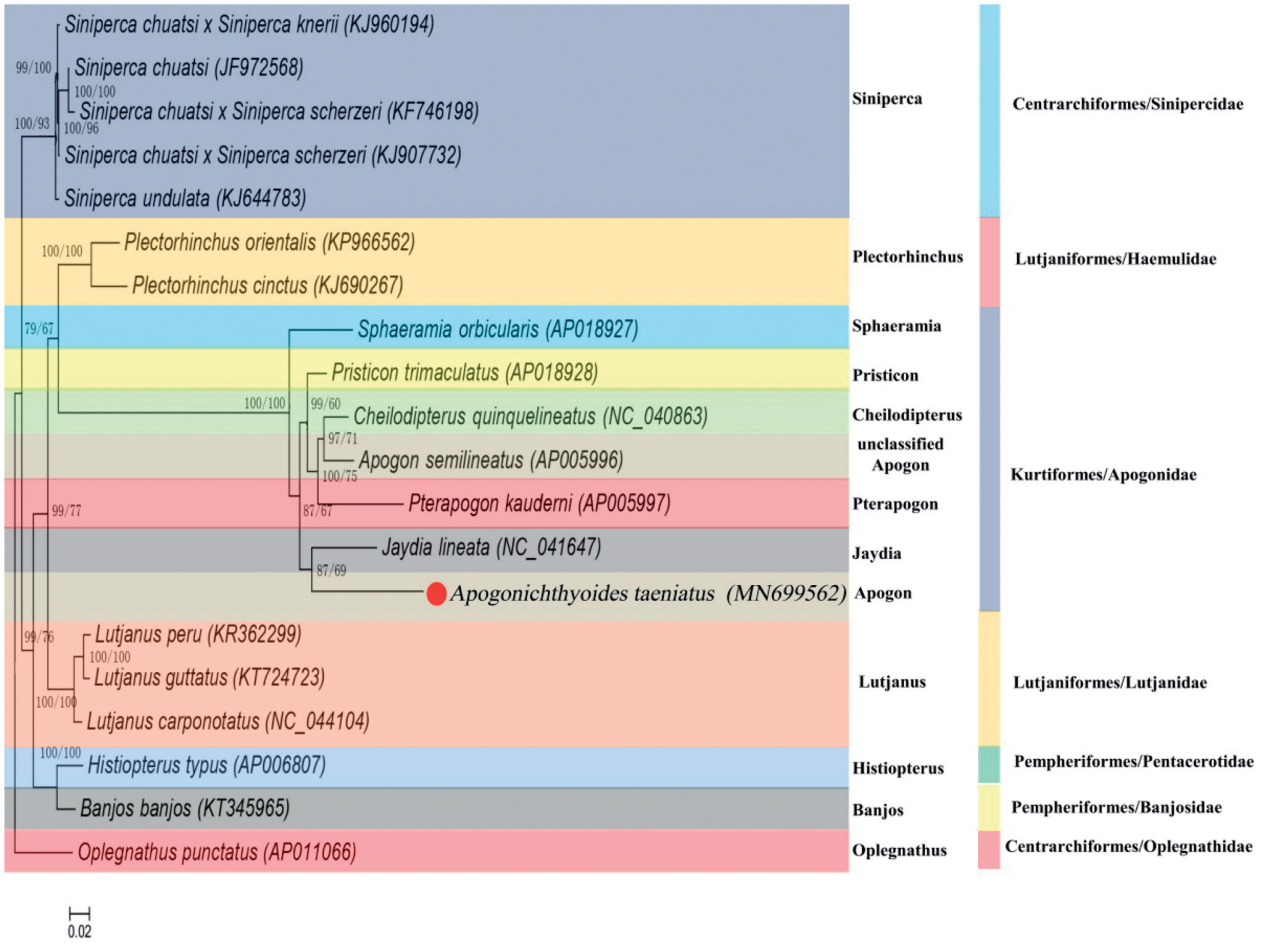
Phylogenetic tree of *A. taeniatus* relationships from the amino acid datasets. Sequence alignment of 13 PCGs was analyzed using the MEGA 7.0 with ML method. The accession numbers of the sequences used in the phylogenetic analysis are shown in Figure.

## Data Availability

The data that support the findings of this study are openly available in the Genbank of NCBI at https://www.ncbi.nlm.nih.gov/, reference number MN699562.
